# Experiments on Hospital Patients

**Published:** 1901-11-02

**Authors:** 


### THE INSTITUTIONAL WORKSHOP.

### EXPERIMENTS ON HOSPITAL PATIENTS.

For some weeks past the *Times* newspaper has admitted to its columns a correspondence in regard to a much-advertised remedy for, or treatment of, consumption. Curiously enough, perhaps because the writer who started the correspondence expressed a willingness to share the expense of testing this method to the extent of £1,000, and so gave a well-to-do air of respectability to the suggestion, several medical men of good standing intervened in the matter. Their letters, of course, went to show the absurdity of the proposals. In fact, the whole affair has been so ridiculous that we have hitherto taken no notice of it, and our only reason for referring to it now is because of the extraordinary views some people seem to have in regard to the responsibility of hospital managers and hospital physicians to their patients. Whatever might be the case were the hospitals rate-supported institutions—when perhaps popular clamour and political influences might be expected to have a certain sway—the managers of the existing voluntary hospitals stand in the position of trustees, receiving vast sums of money from the public "in trust," as the lawyers would say, to expend it to the best advantage in the medical treatment of the sick poor. Nowhere in the constitution of the voluntary hospitals is there anything to be found which would justify their managers in handing over the patients in their wards to be submitted to experimental treatment, however impudent the claims of the method or however lavish its supporters might be in their offers of money. It is no small matter to obtain an appointment on the staff of a great London hospital. The governors of these hospitals exercise the most jealous care in the selection of the practitioners to whom the treatment of the sick under their charge is to be entrusted, and those who are appointed are in honour bound to use no treatment on their patients except what they personally believe to be to their patients' advantage. Yet there are people who do not seem to see this, and who apparently think it the simplest matter in the world to hand over a wardful of sick folk to be experimented on by any outsider who can bring a few testimonials as to the success of his "remedies." In a letter in the *Times* last week it is actually brought up as a matter of complaint that when a certain Mr. Alabone, years ago, made application to the authorities at Brompton Hospital to place a ward at his disposal for the demonstration of his treatment, instead of their accepting the suggestion, an answer was given that if full particulars and details were furnished the treatment would be examined into by the medical officers, who would report on the result. It appears that on the inventor of this "treatment" declining to accept these conditions nothing more was done in the matter. And this is what this much-aggrieved writer speaks of as the "suppression of the treatment." To us it seems an admirable testimonial to the hospital—another proof, if proof were needed, that the voluntary hospitals of London are not devoted to experiments, as so many penny-a-liners pretend to think they are, but are places in which the treatment of the patients is carried out on the same lines (only often in a much more complete and perfect manner) as those which are followed in private practice. This is the strong position held by the voluntary hospitals, and when irresponsible people, in a light and airy manner, after their wont, bring it as a charge against hospital managers and hospital physicians that they have declined to place wards at the disposal of any Dick, Tom, or Harry (often advertising people with their own little axes to grind and their own little fortunes to make), who may request the loan of a few patients so that they may try their precious experiments on them, let us remember that these managers and these physicians are but fulfilling their trust in refusing to submit their patients to such experiments, and in doing to them, in regard to treatment, as they themselves would be done by.

